# Supplementary figures and images for: Retention of lumpy skin disease virus in Stomoxys spp (Stomoxys calcitrans, Stomoxys sitiens, Stomoxys indica) following intrathoracic inoculation, Diptera: Muscidae
Source: PLoS One. 2021 Feb 19;16(2):e0238210. doi: 10.1371/journal.pone.0238210 (PMC7894917; doi:10.1371/journal.pone.0238210)

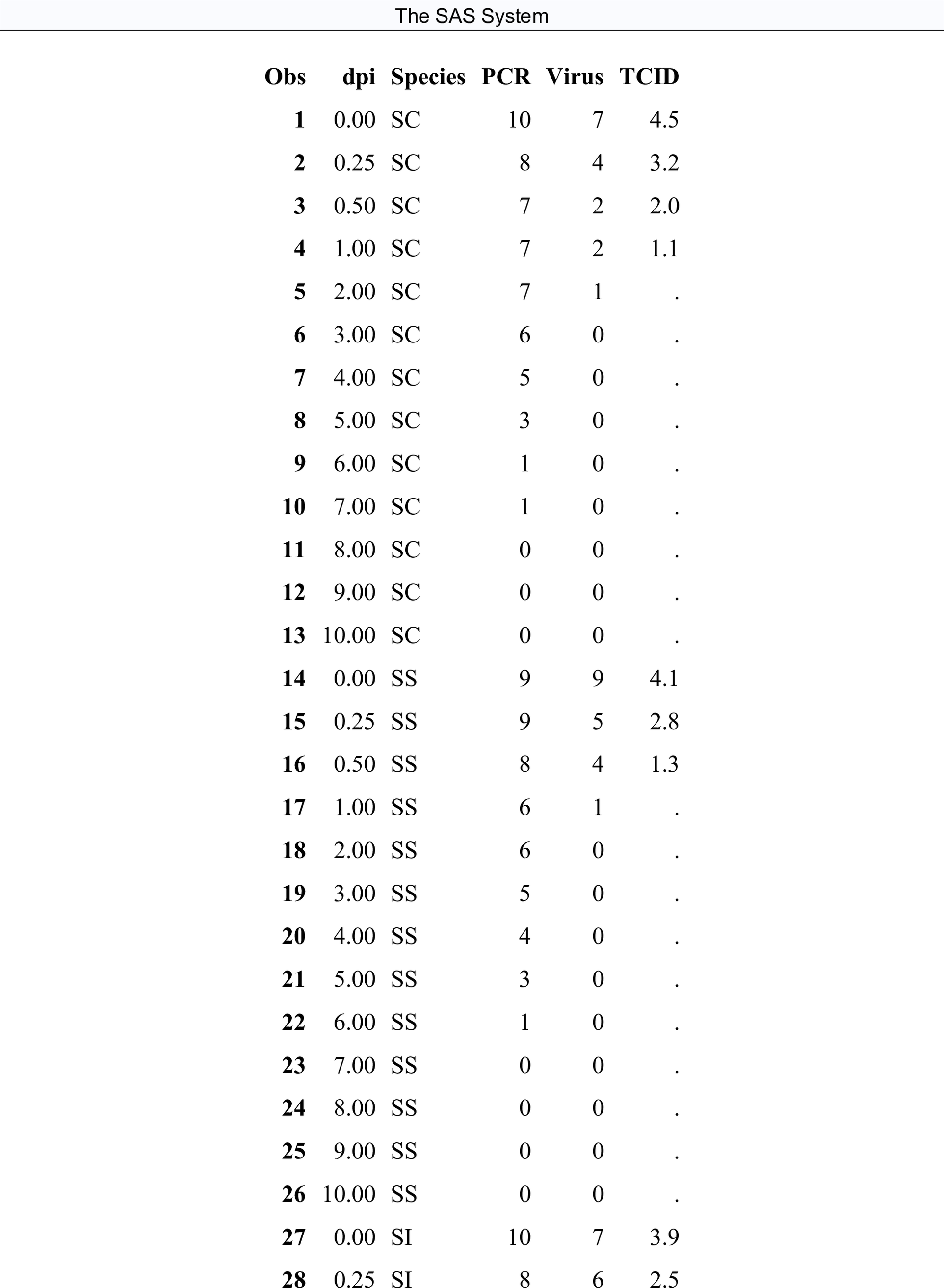

Supplement: S1 Fig — (ZIP) [file pone.0238210.s001.zip › PACE Corrected/LSD output.tif]

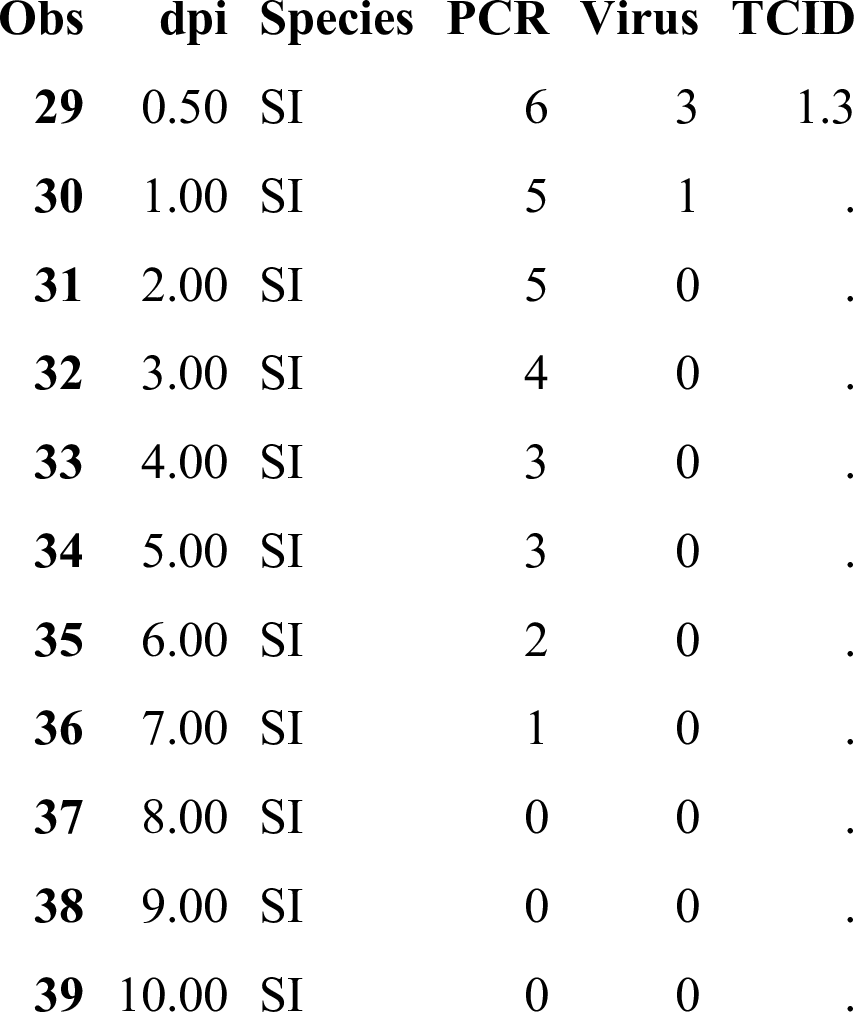

Supplement: S1 Fig — (ZIP) [file pone.0238210.s001.zip › PACE Corrected/LSD output.tif]

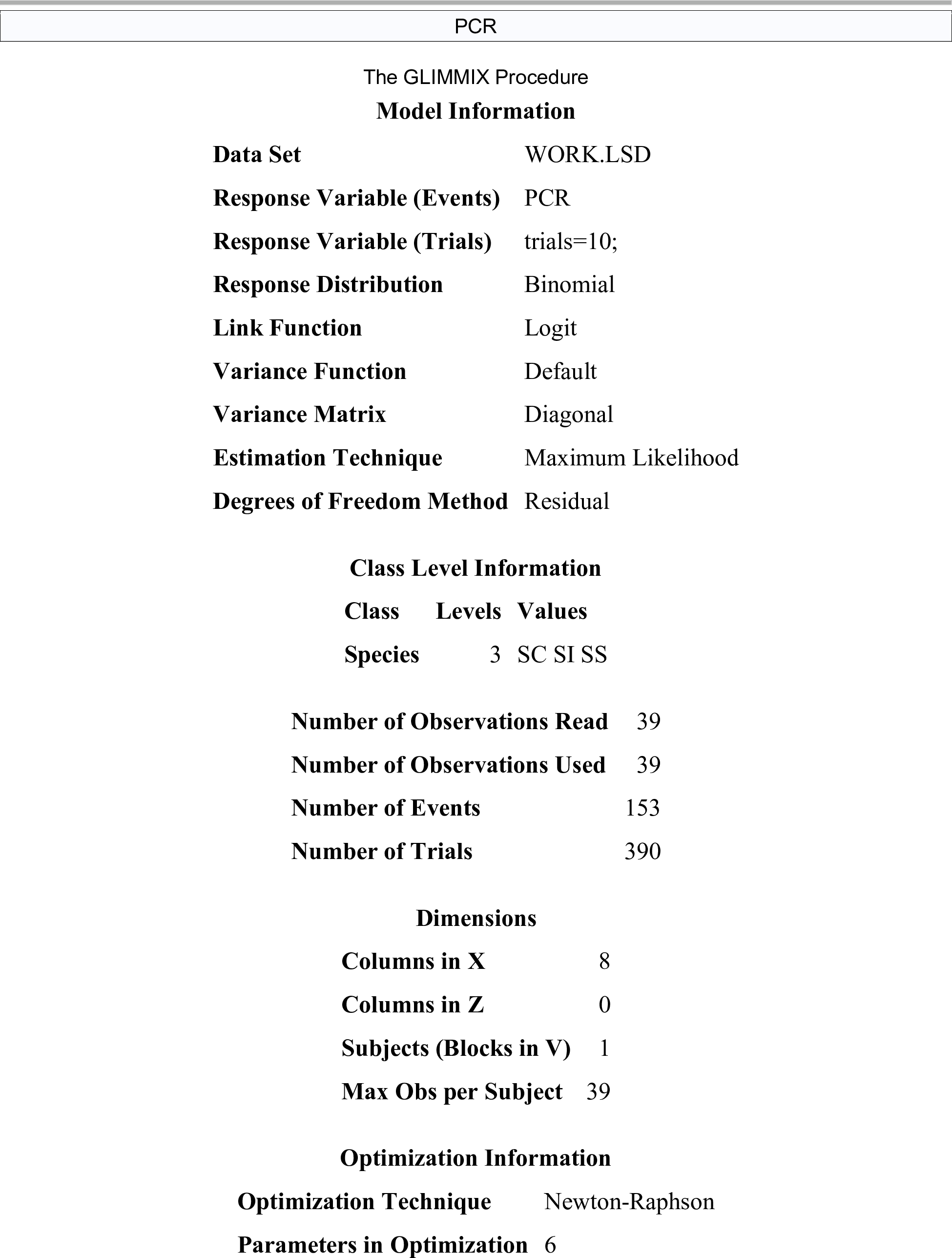

Supplement: S1 Fig — (ZIP) [file pone.0238210.s001.zip › PACE Corrected/LSD output.tif]

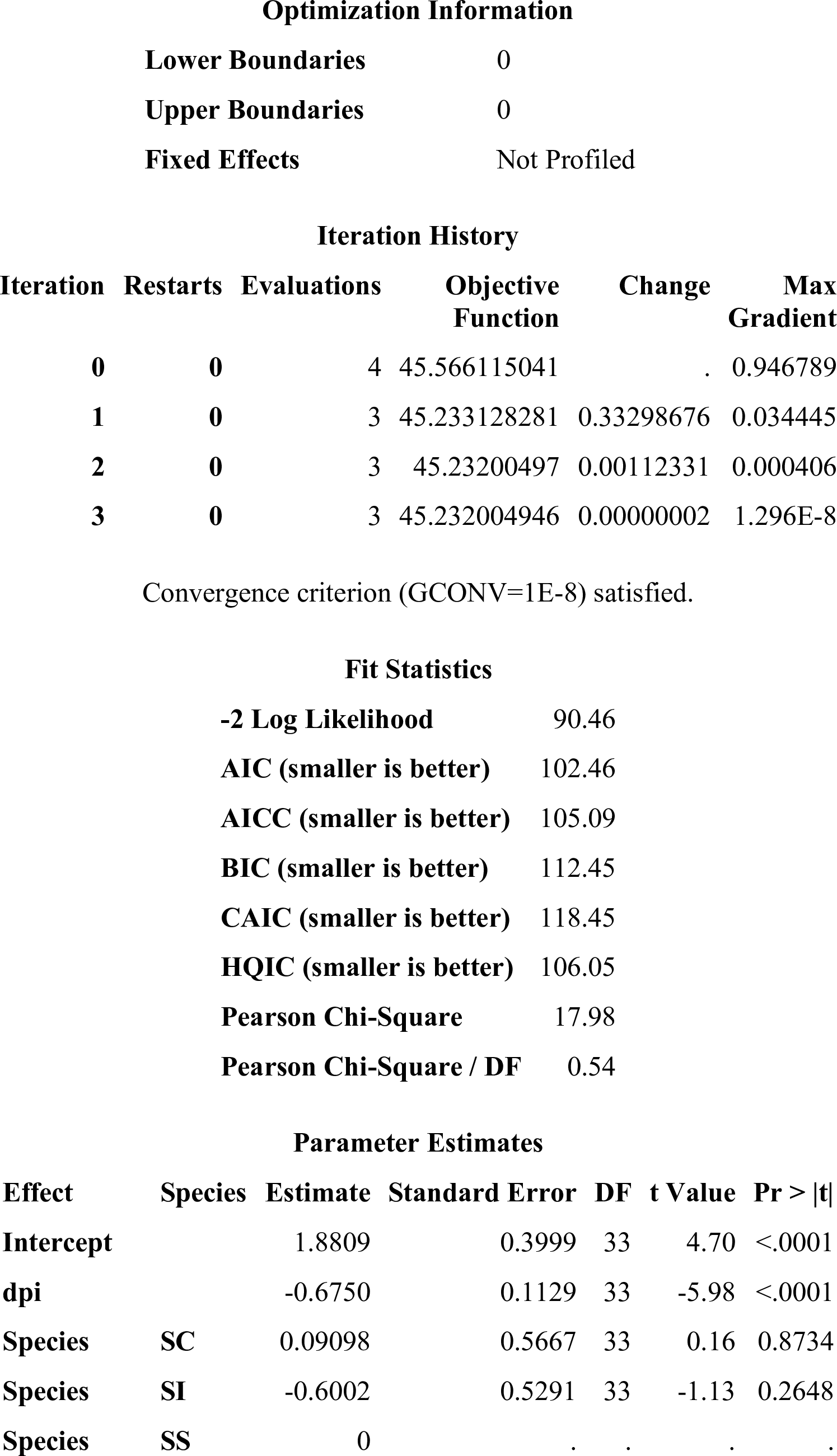

Supplement: S1 Fig — (ZIP) [file pone.0238210.s001.zip › PACE Corrected/LSD output.tif]

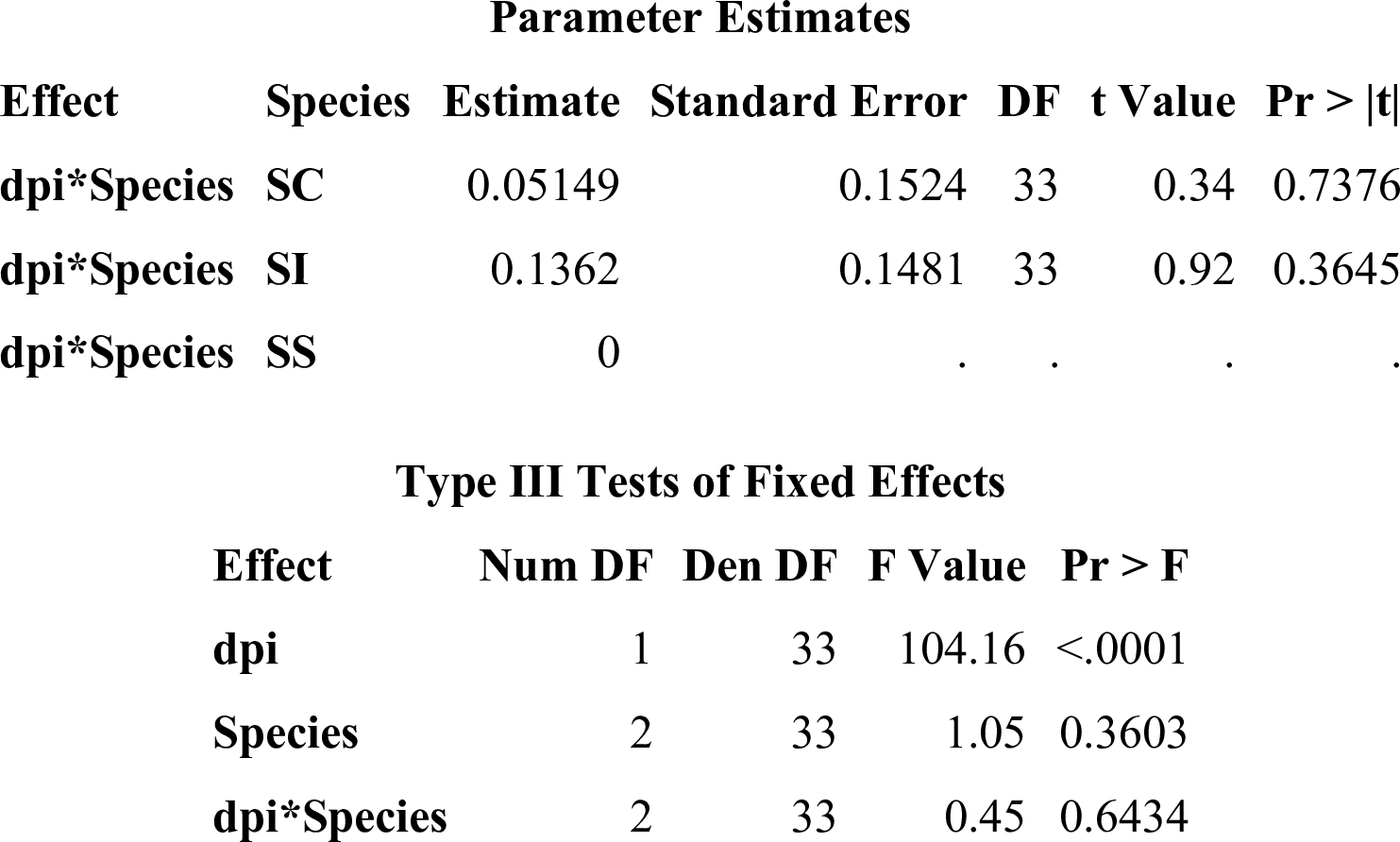

Supplement: S1 Fig — (ZIP) [file pone.0238210.s001.zip › PACE Corrected/LSD output.tif]

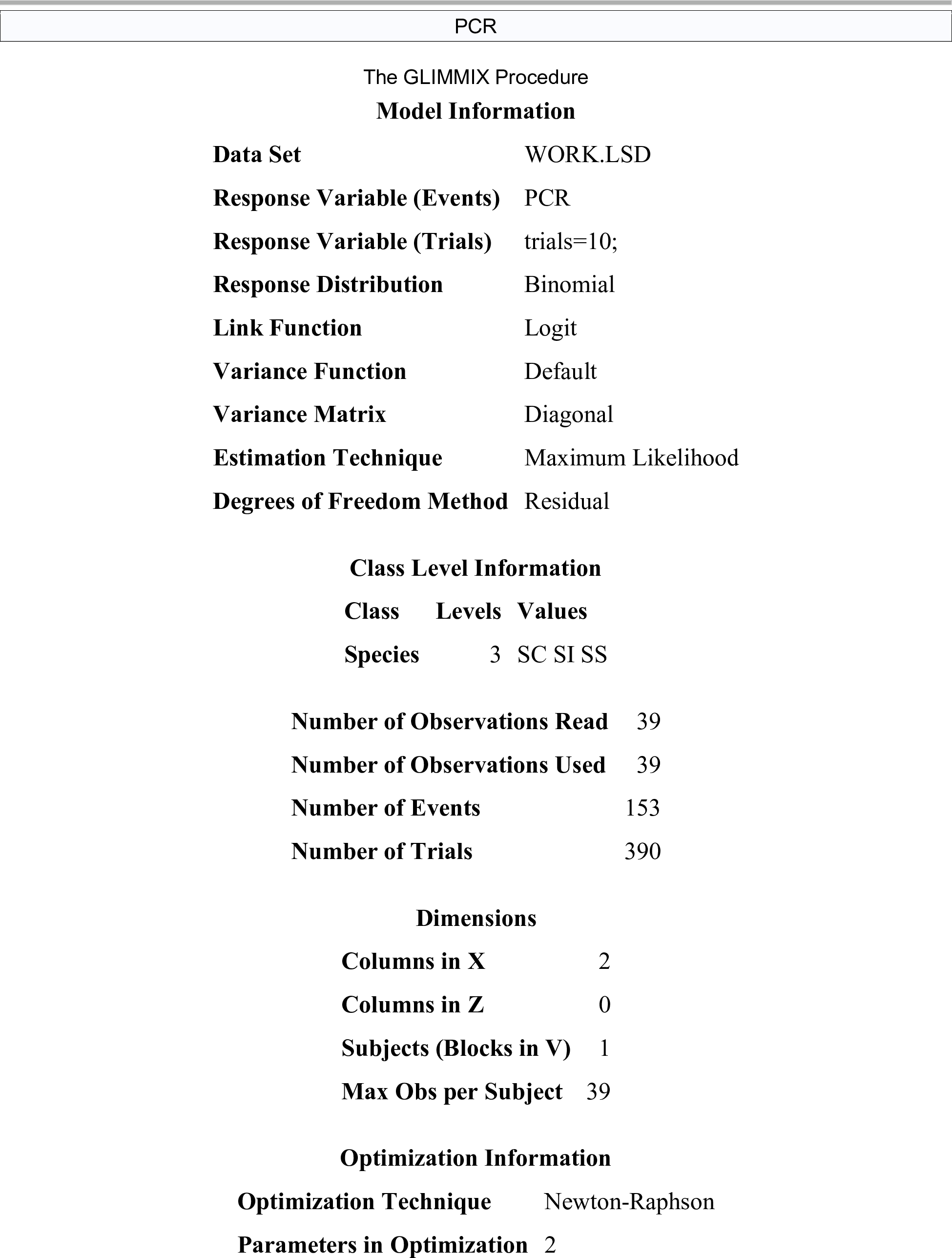

Supplement: S1 Fig — (ZIP) [file pone.0238210.s001.zip › PACE Corrected/LSD output.tif]

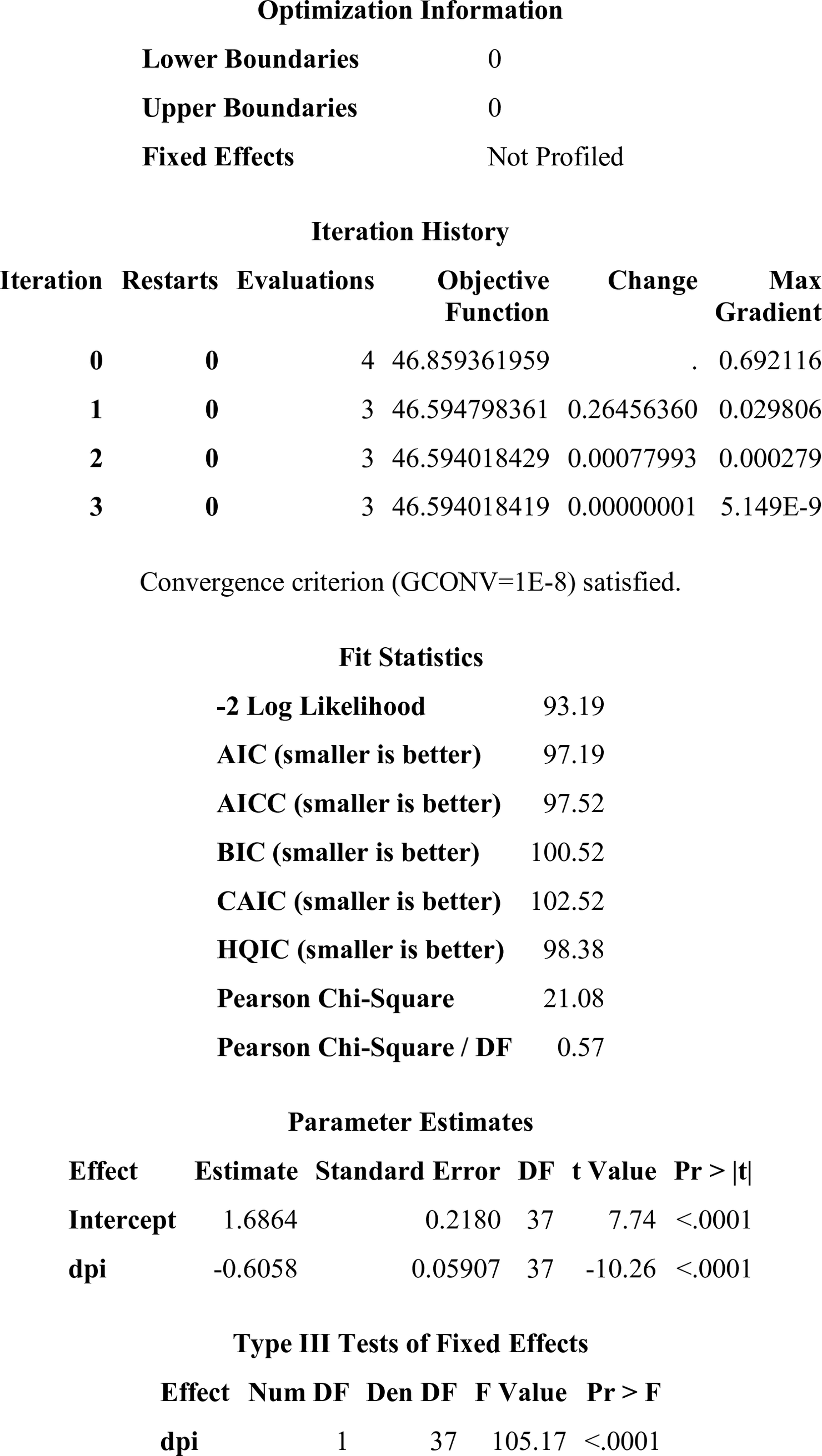

Supplement: S1 Fig — (ZIP) [file pone.0238210.s001.zip › PACE Corrected/LSD output.tif]

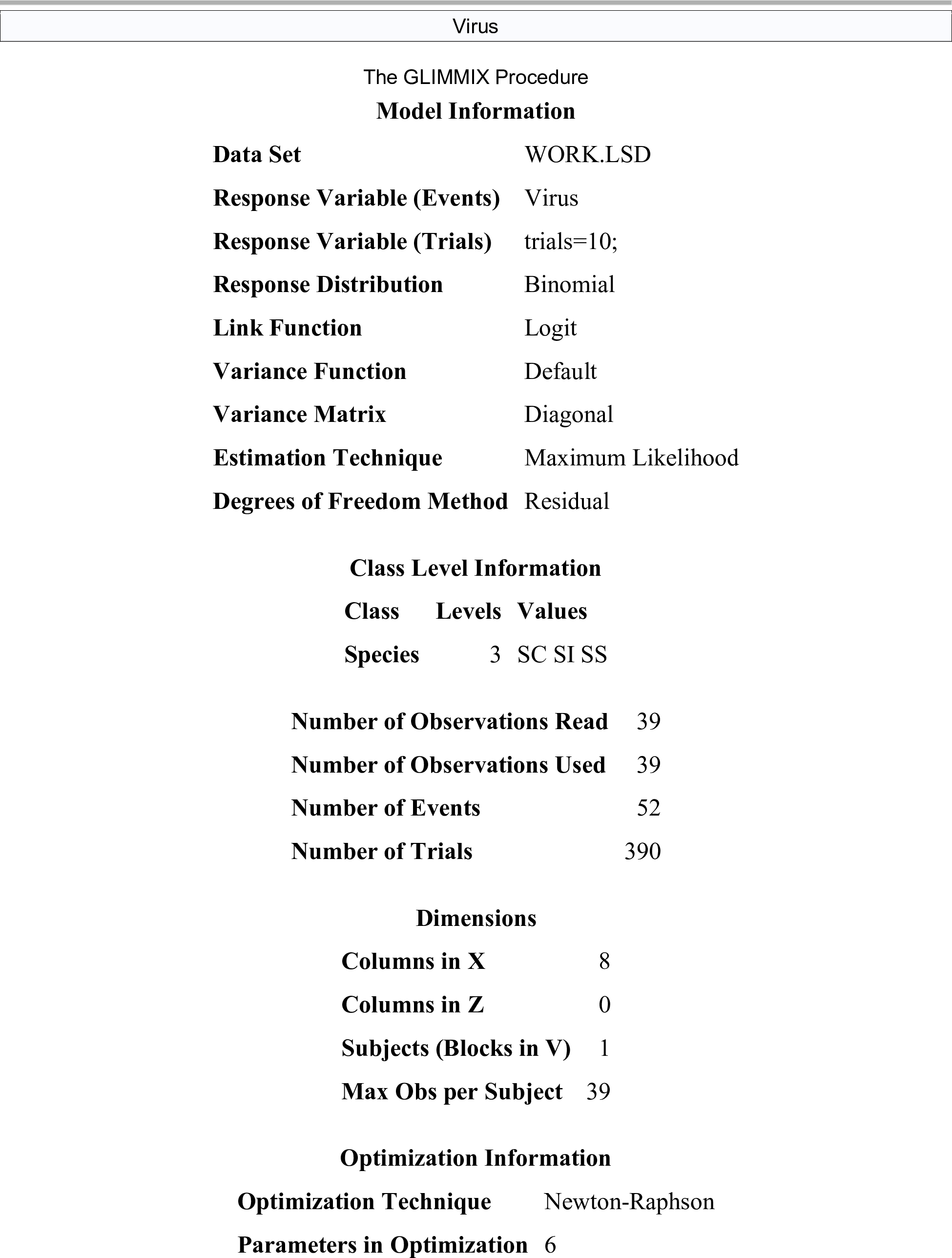

Supplement: S1 Fig — (ZIP) [file pone.0238210.s001.zip › PACE Corrected/LSD output.tif]

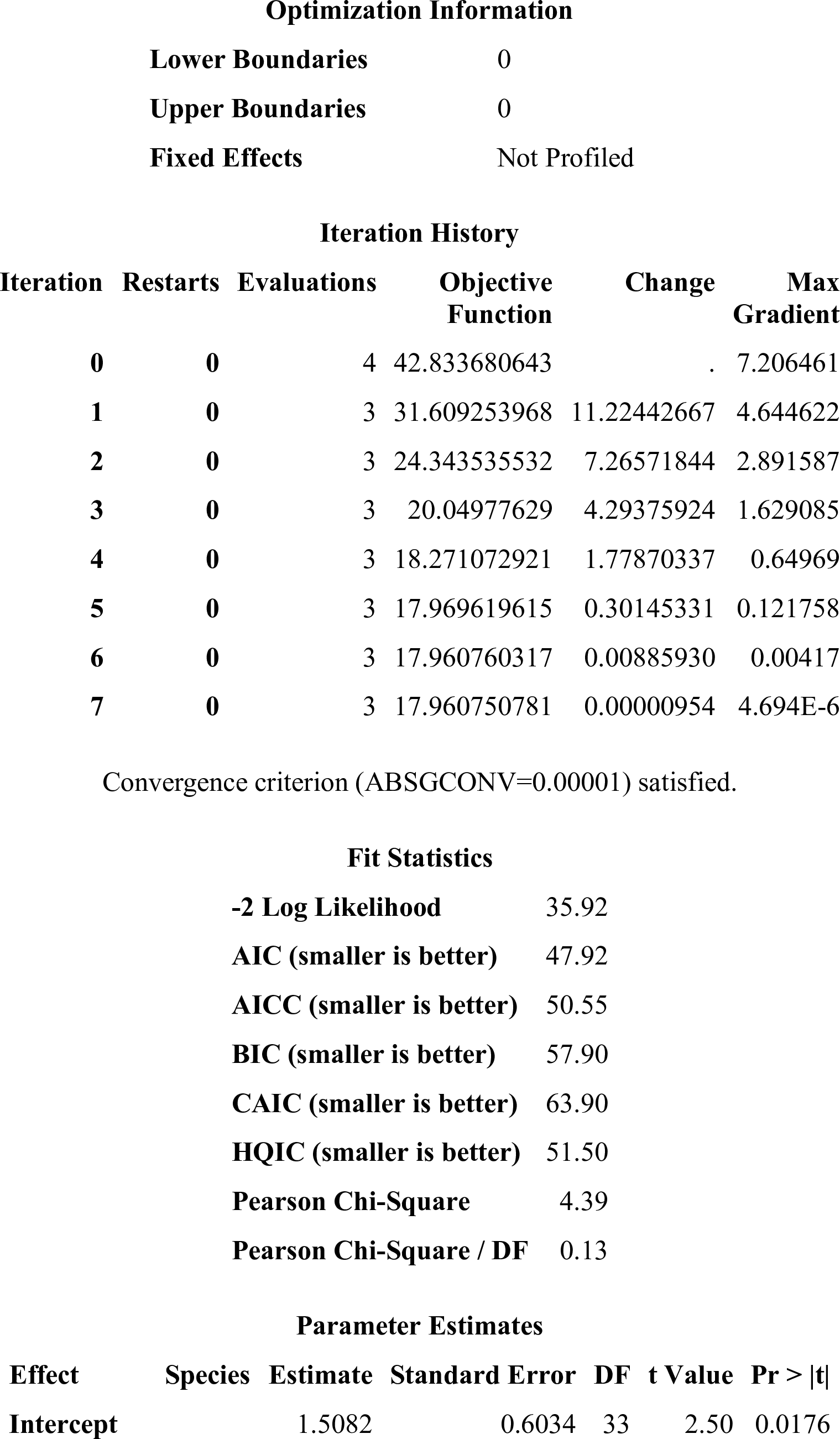

Supplement: S1 Fig — (ZIP) [file pone.0238210.s001.zip › PACE Corrected/LSD output.tif]

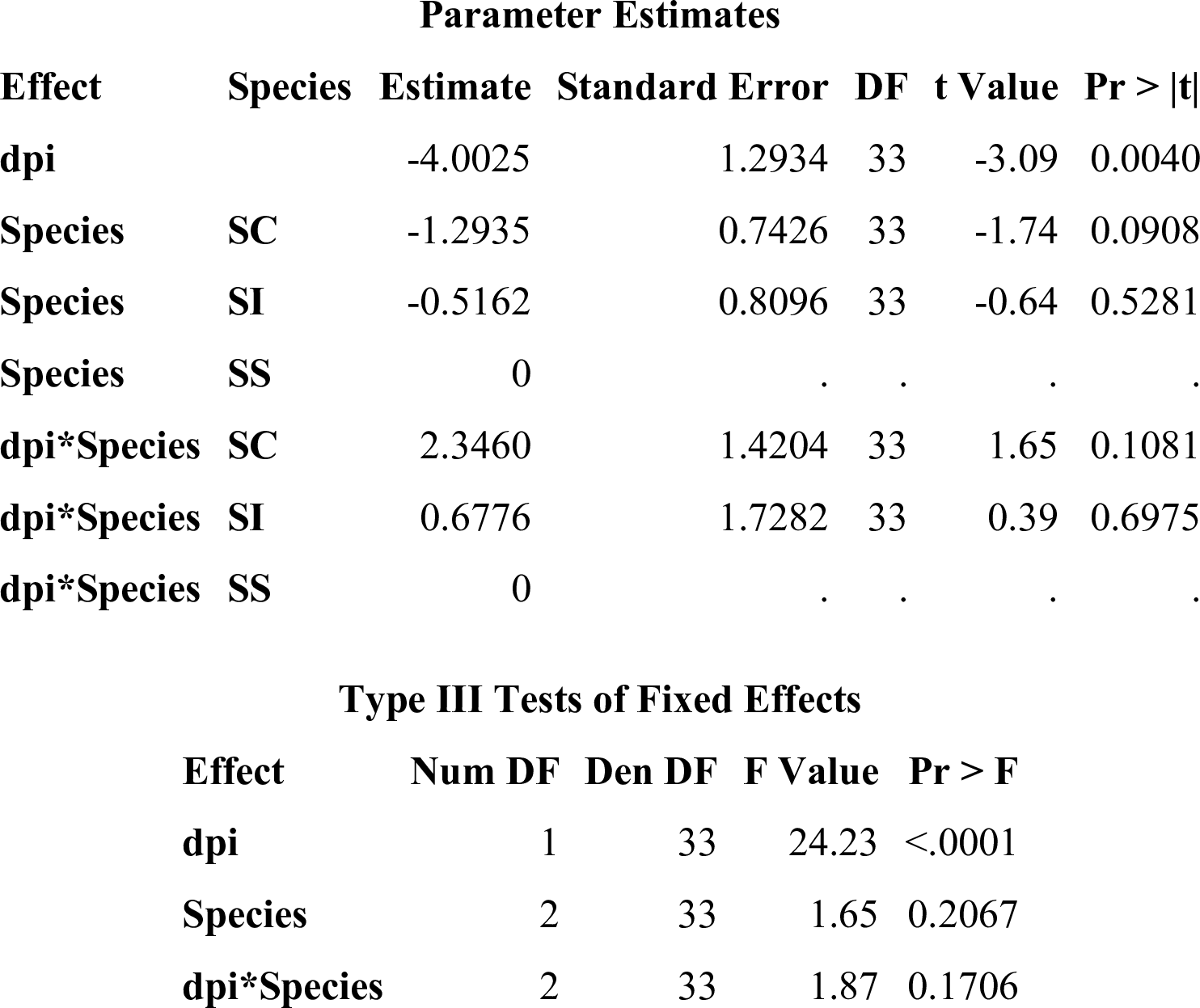

Supplement: S1 Fig — (ZIP) [file pone.0238210.s001.zip › PACE Corrected/LSD output.tif]

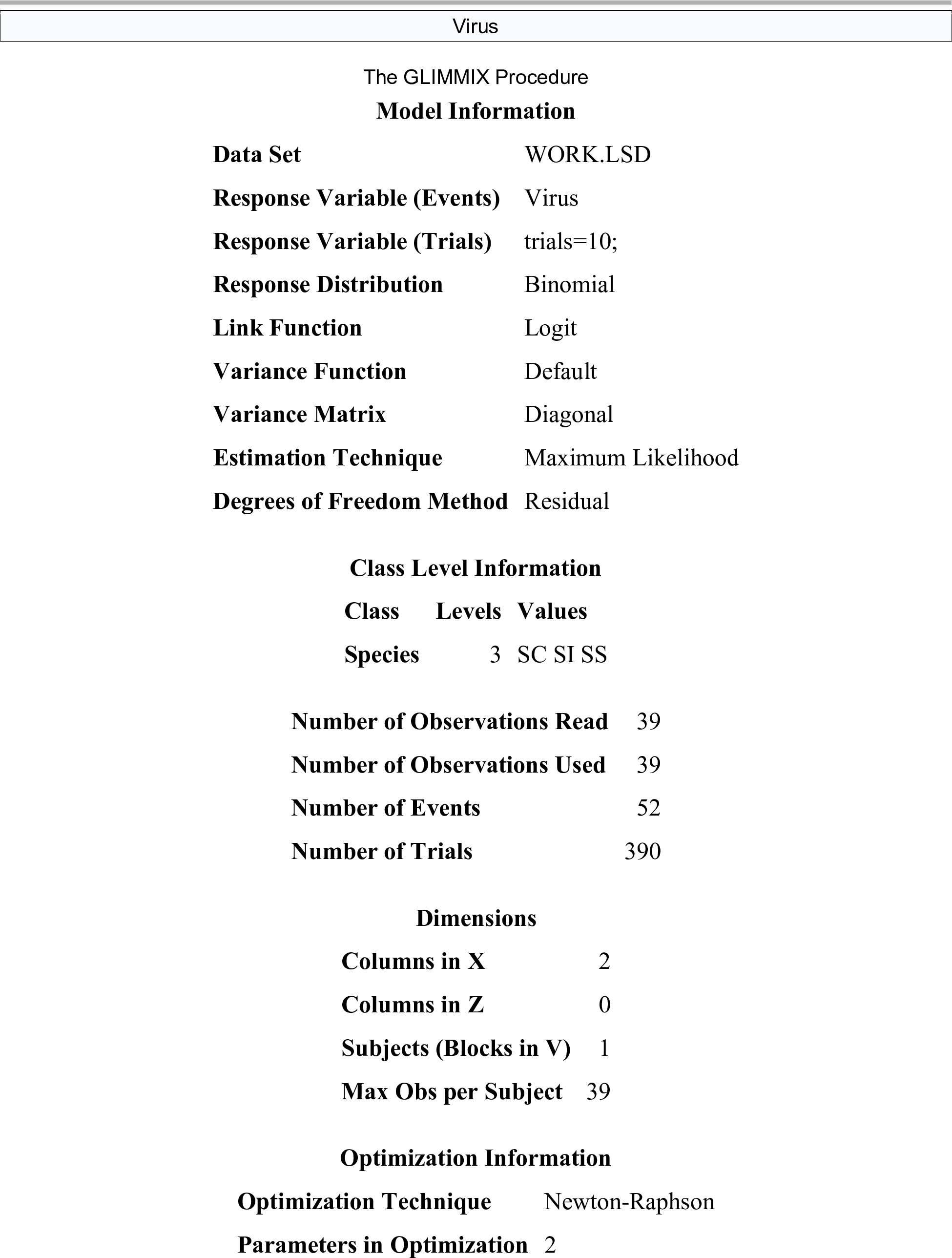

Supplement: S1 Fig — (ZIP) [file pone.0238210.s001.zip › PACE Corrected/LSD output.tif]

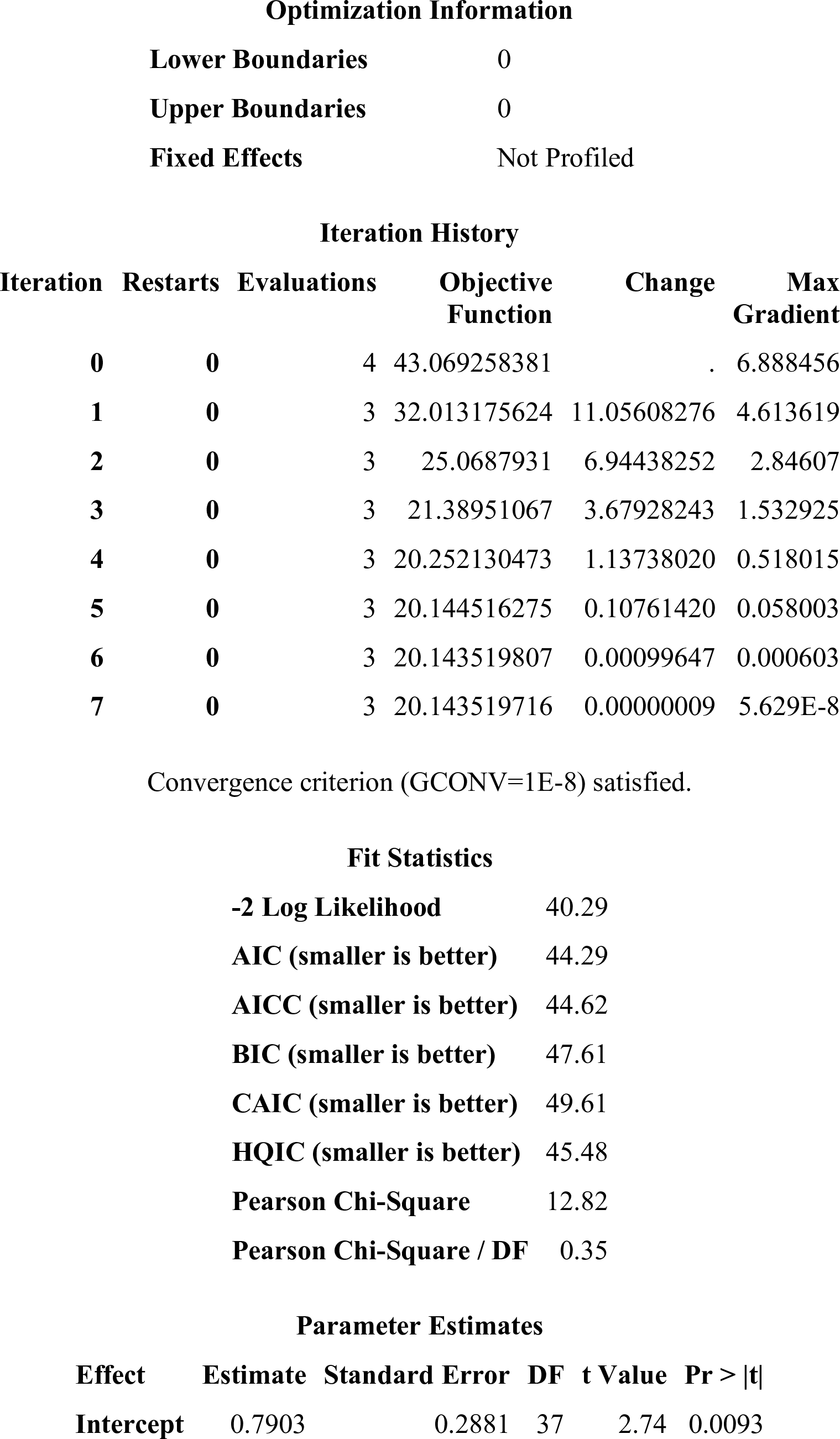

Supplement: S1 Fig — (ZIP) [file pone.0238210.s001.zip › PACE Corrected/LSD output.tif]

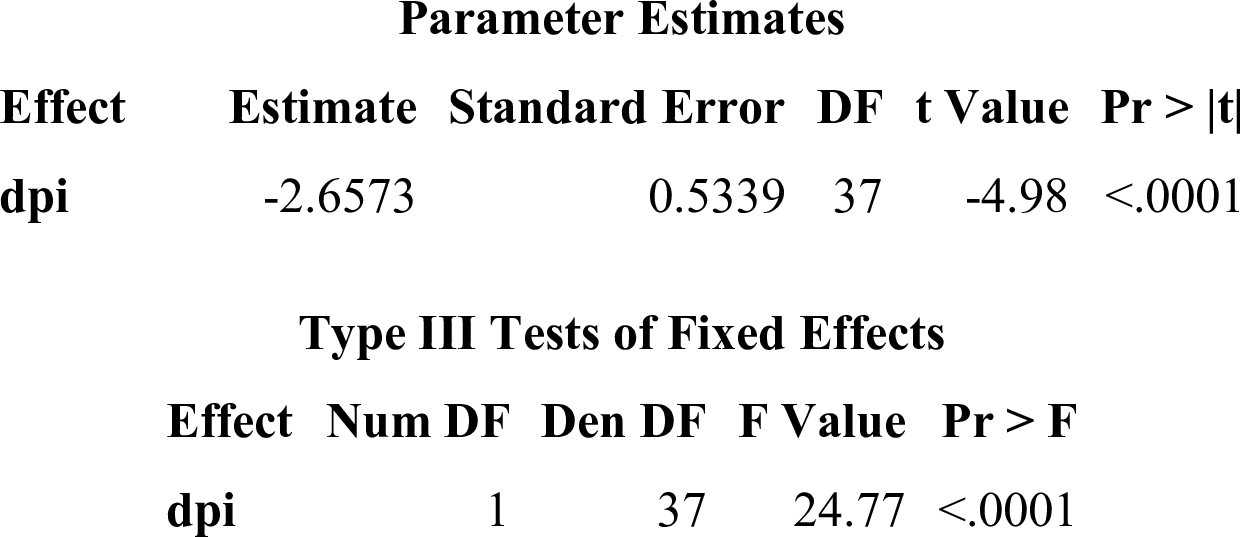

Supplement: S1 Fig — (ZIP) [file pone.0238210.s001.zip › PACE Corrected/LSD output.tif]

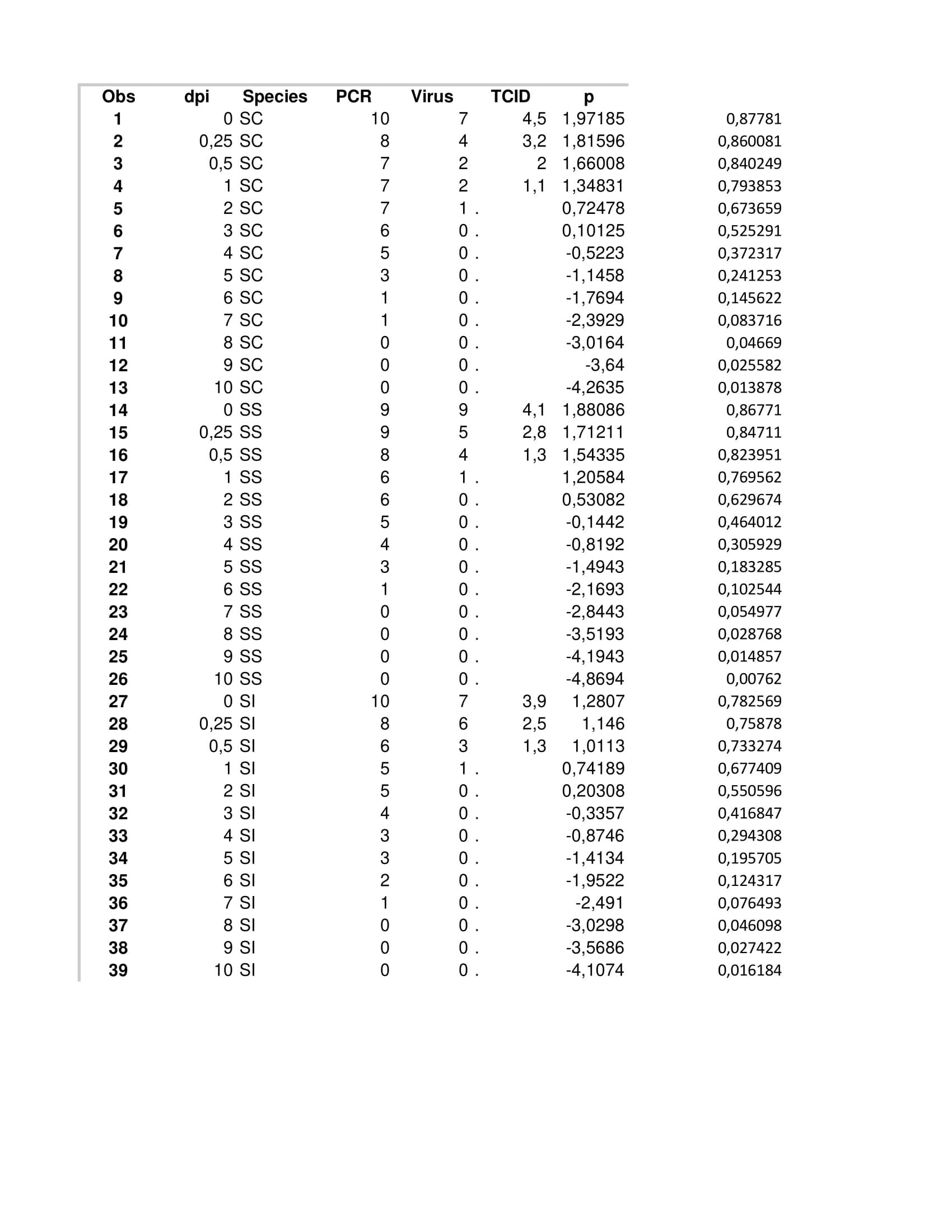

Supplement: S1 File — (ZIP) [file pone.0238210.s002.zip › LSD_0003.tif]

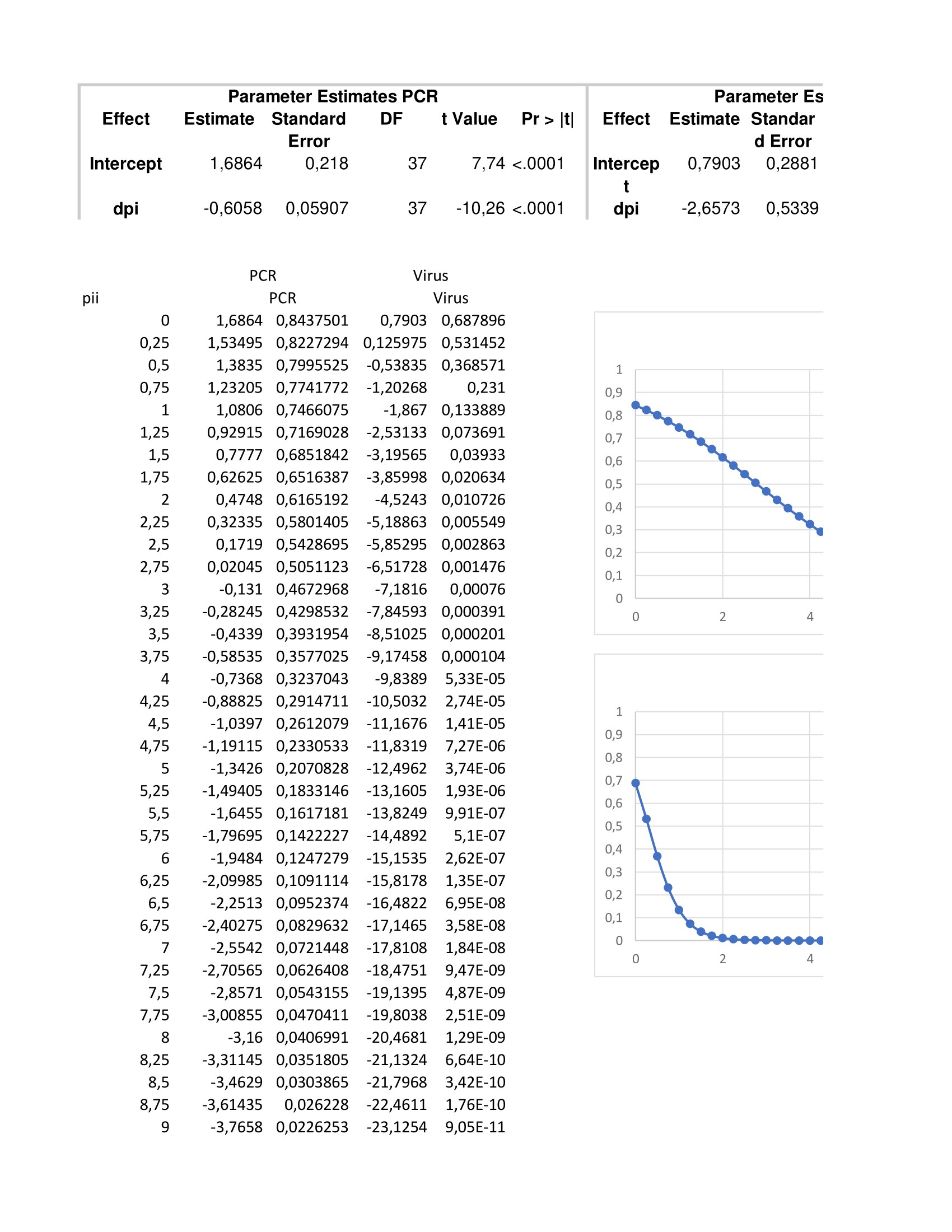

Supplement: S1 File — (ZIP) [file pone.0238210.s002.zip › LSD_0005.tif]

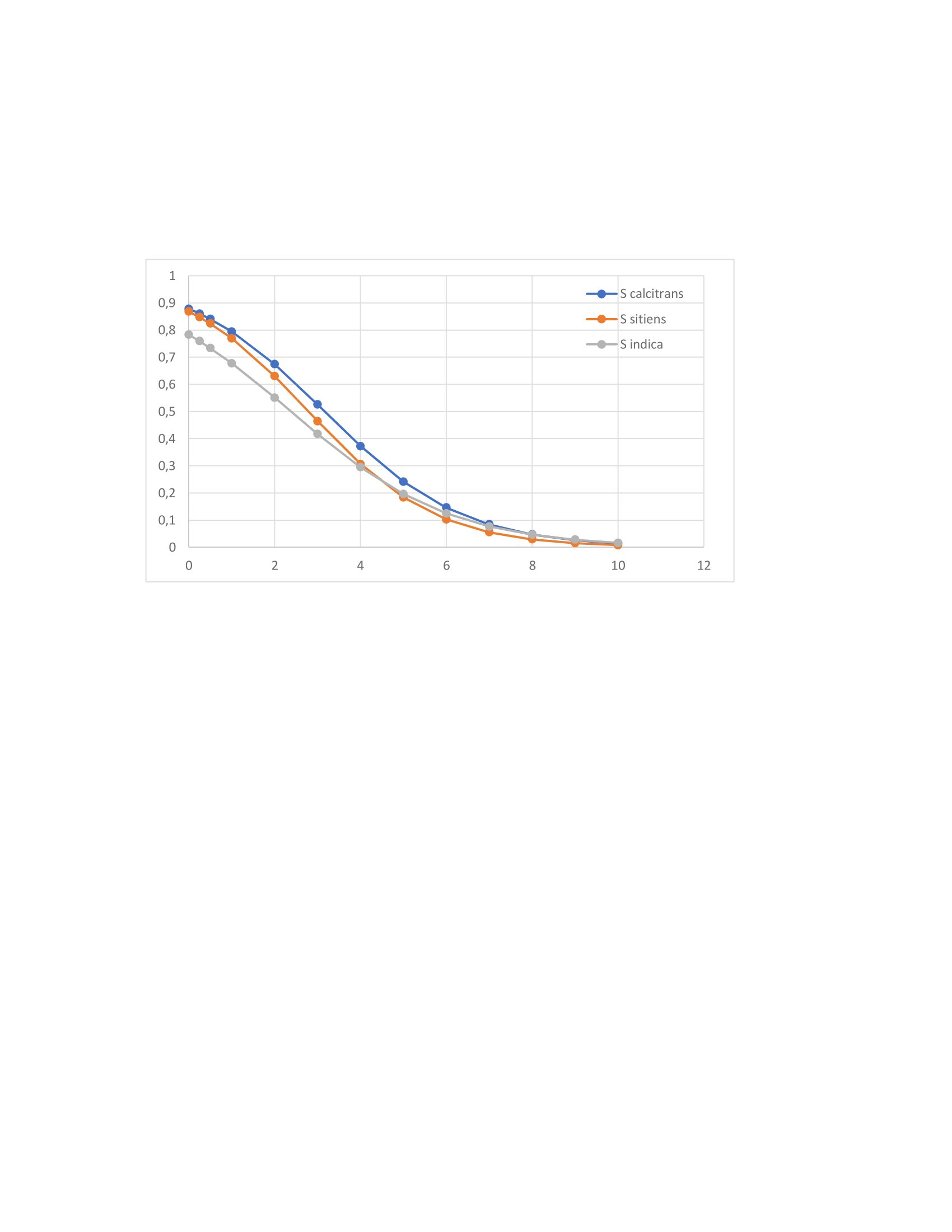

Supplement: S1 File — (ZIP) [file pone.0238210.s002.zip › LSD_0004.tif]

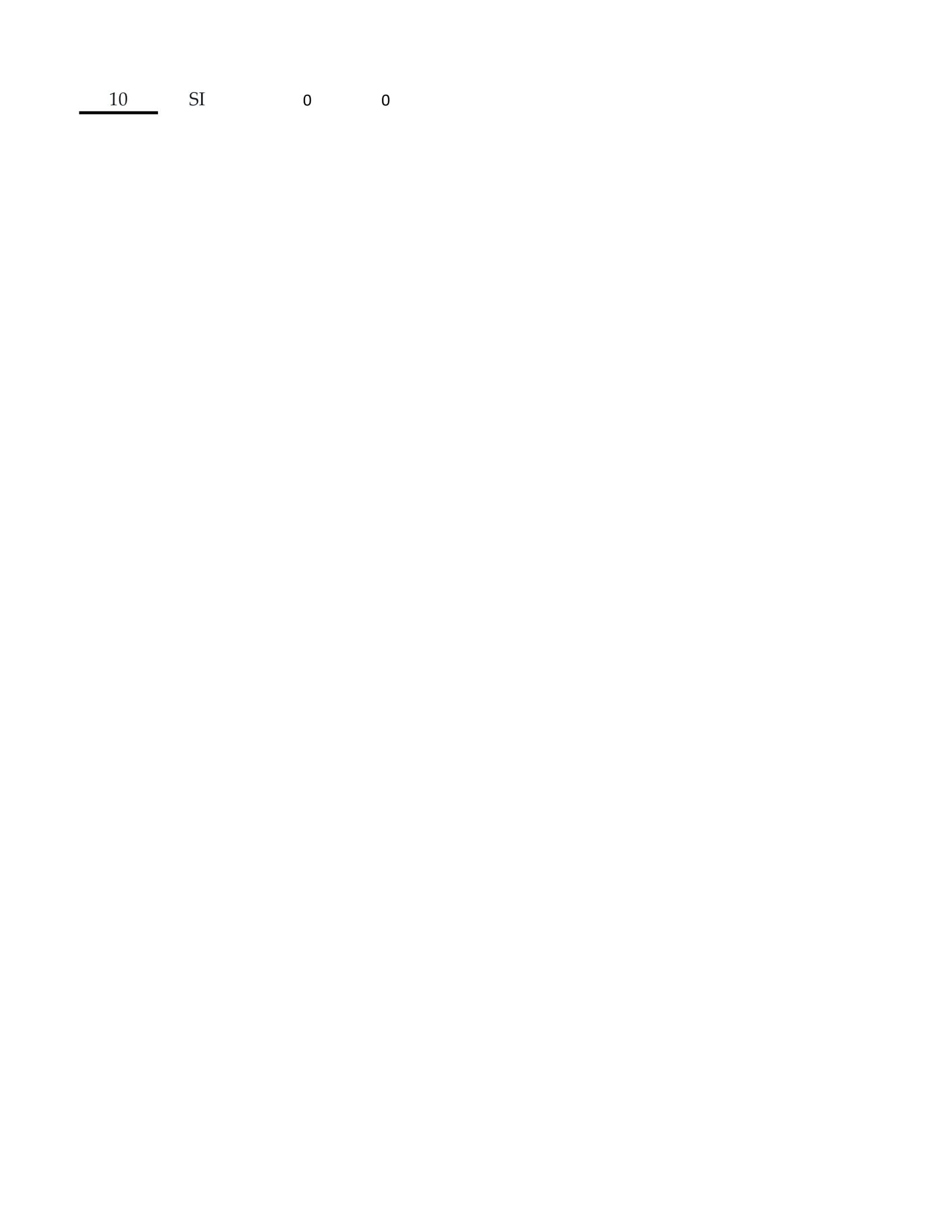

Supplement: S1 File — (ZIP) [file pone.0238210.s002.zip › LSD_0002.tif]

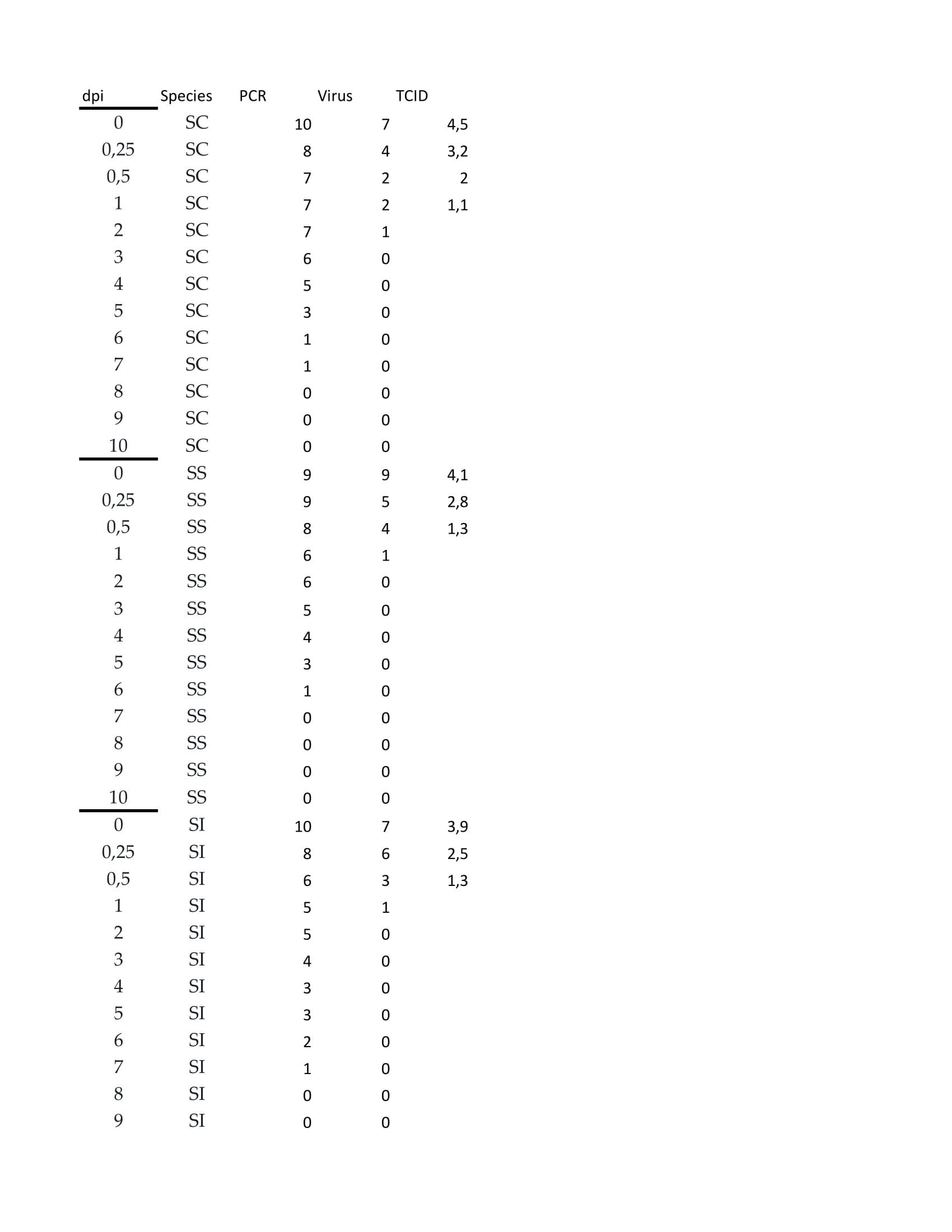

Supplement: S1 File — (ZIP) [file pone.0238210.s002.zip › LSD_0001.tif]

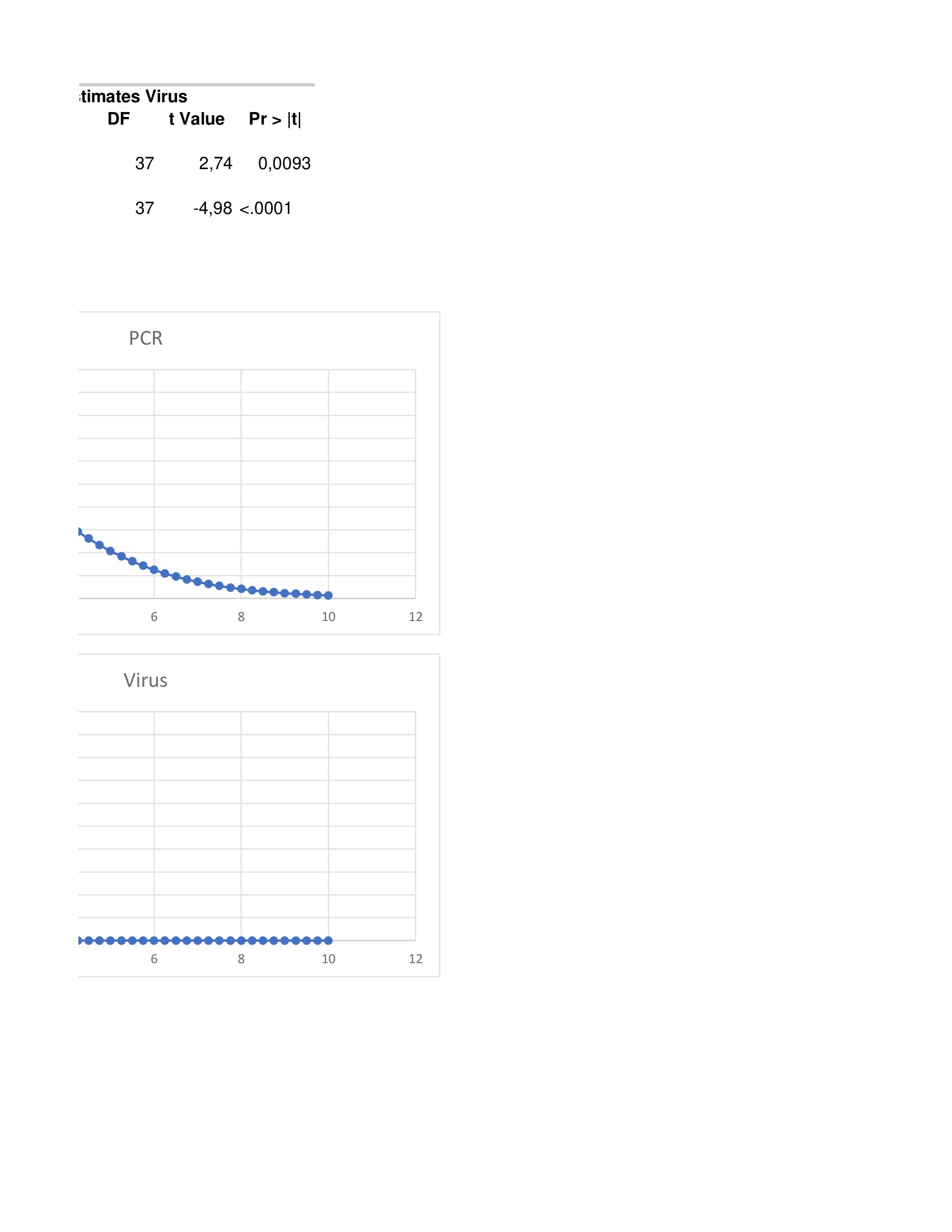

Supplement: S1 File — (ZIP) [file pone.0238210.s002.zip › LSD_0007.tif]

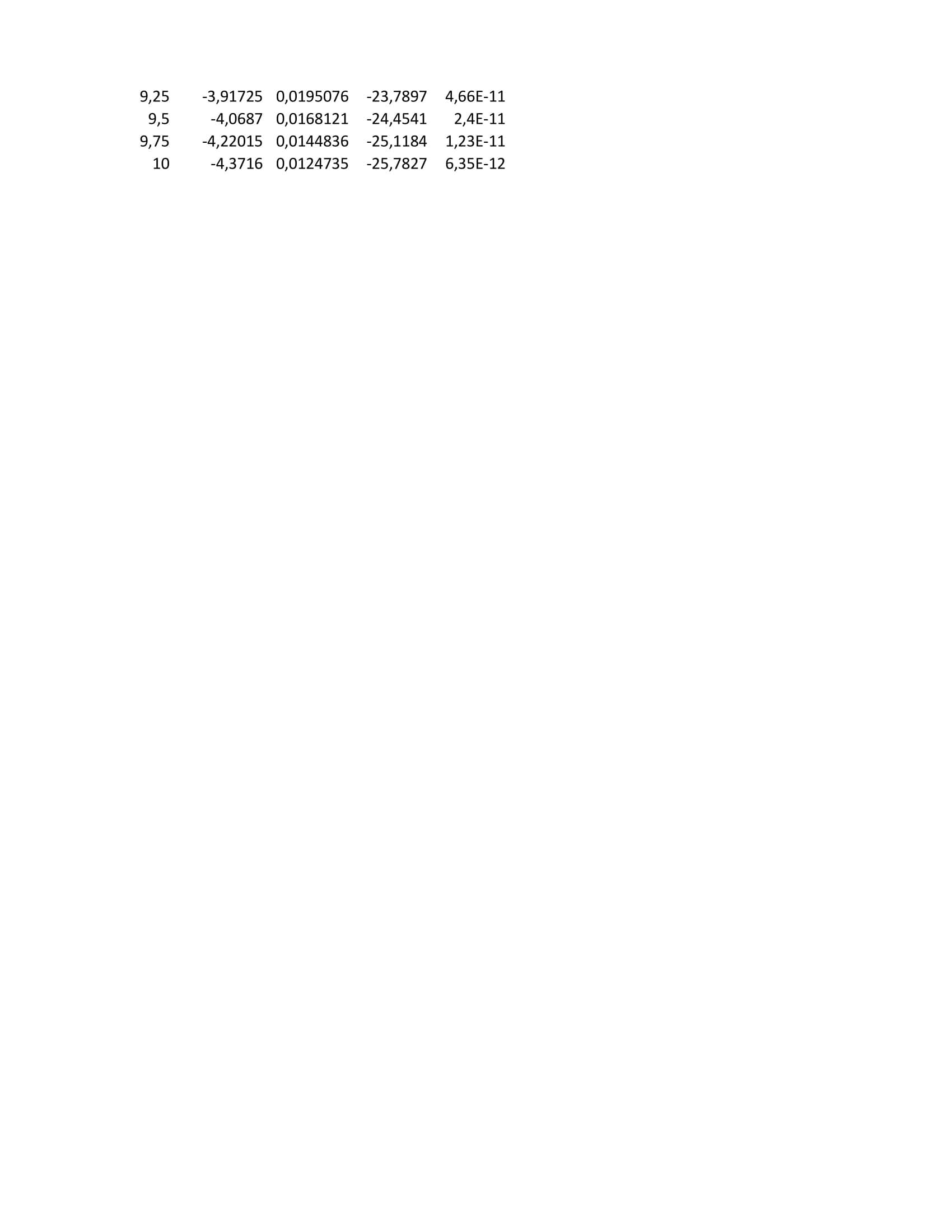

Supplement: S1 File — (ZIP) [file pone.0238210.s002.zip › LSD_0006.tif]
