# Supplementary material for: Retention of lumpy skin disease virus in Stomoxys spp (Stomoxys calcitrans, Stomoxys sitiens, Stomoxys indica) following intrathoracic inoculation, Diptera: Muscidae
Source: PLoS One. 2021 Feb 19;16(2):e0238210. doi: 10.1371/journal.pone.0238210 (PMC7894917; doi:10.1371/journal.pone.0238210)
Supplement: Attachment — Submitted filename: Comments LSDV Stomoxys spp.docx [file pone.0238210.s003.docx]

The authors describe intrathoracic inoculation of three different Stomoxys species with LSDV, to determine retention of LSDV in the hemolymph of these insects. Therefore, they used gel-based PCR assay and virus isolation at different time points after inoculation.

**General:**

- Be consistent during the whole manuscript by spelling *Stomoxys calcitrans, Stomoxys*
- *sitiens, Stomoxys indica*.
- English language: minor improvement necessary

**Introduction:**

- Could be more references for clinical signs, e.g. Babiuk et al., which is cited later
- Mechanical transmission of LSDV was also demonstrated by Chihota et al. in 2001 and 2003
- L. 43: better “these studies” than “their study”
- L. 45: Stomoxys in italic
- Define the aims of the study in more detail, e.g. why intrathoracic inoculation instead of spiked blood meals? Why is it important to bypass the midgut barrier in this study concept?

**Material & Methods:**

- L 72: 10^6^ TCID_50_ per ml??
- Three times passaged in vitro using calves – please explain this
- After how many hours/days were the individual control flies tested?
- Was a pool of flies always 10 flies/pool or only at 0h?
- Pools of flies were homogenized in 300 µl Hanks solution, but 1 mL of suspension was used for virus isolation – how was the volume increased?

**Results:**

- Again, when were the individual control flies tested?
- L 119: Pool samples
- L 131: at which stage?

**Discussion:**

- Decrease of titer is in contrast to Rochon, Baker, who, due to the reference cited, examined Porcine Reproductive and Respiratory Syndrome Virus. This should be clarified in the text.
- Use of “in this study” is misleading when describing the present study
- Reference for impact and description of the mentioned impact of the alimentary tract on LSDV is missing
- Again: Schurrer, Dee also examined porcine reproductive and respiratory syndrome virus, this should be mentioned to be clear
