# Supplementary material for: Retention of lumpy skin disease virus in Stomoxys spp (Stomoxys calcitrans, Stomoxys sitiens, Stomoxys indica) following intrathoracic inoculation, Diptera: Muscidae
Source: PLoS One. 2021 Feb 19;16(2):e0238210. doi: 10.1371/journal.pone.0238210 (PMC7894917; doi:10.1371/journal.pone.0238210)
Supplement: Attachment — Submitted filename: Response to Reviewers.docx [file pone.0238210.s004.docx]

Response to Reviewers 1 and 2 Comments

**Point 1:** Be consistent during the whole manuscript by spelling *Stomoxys calcitrans, Stomoxys sitiens, Stomoxys indica*.

**Response 1:** Revised as requested

**Point 2:** English language: minor improvement necessary

**Response 2:** Revised as requested

**Point 3:** Could be more references for clinical signs, e.g. Babiuk et al., which is cited later

**Response 3:** Additional references are added

**Point 4:** Mechanical transmission of LSDV was also demonstrated by Chihota et al. in 2001 and 2003

**Response 4:** References are cited

**Point 5:** L. 43: better “these studies” than “their study”

**Response 5:** Revised as requested.

**Point 6:** Stomoxys in italic

**Response 6:** Revised as requested.

**Point 7:** Define the aims of the study in more detail, e.g. why intrathoracic inoculation instead of spiked blood meals? Why is it important to bypass the midgut barrier in this study concept?

**Response 7:** In blood-feeding insects, the midgut barrier is a significant line of defense against infection as most of the pathogens are ingested orally via contaminated bloodmeal. In the present study, the midgut barrier bypassing procedure was designated to find out LSDV potential to replicate in flies’ hemolymph. If replication occurred, we could state that Stomoxys species can play an important role as a potential LSDV reservoir during the interepizootic period.

**Point 8:** L 72: 10^6^ TCID_50_ per ml??

**Response 8:** yes, 10^6^ TCID_50_/ml (per ml)

**Point 9:** Three times passaged in vitro using calves – please explain this

**Response 9:** This implies that LSDV was passaged three times using alive young bovine animal to make a virus more virulent.

**Point 10:** After how many hours/days were the individual control flies tested?

**Response 10:** 2 hours after inoculation

**Point 11:** Was a pool of flies always 10 flies/pool or only at 0h?

**Response 11:** always 10 flies per pool

**Point 12:** Pools of flies were homogenized in 300 µl Hanks solution, but 1 mL of suspension was used for virus isolation – how was the volume increased?

**Response 12:** There was a misprint. We used 10 µl supernatant to infect cell culture

**Point 13:** Again, when were the individual control flies tested?

**Response 13:** 2 hours after inoculation

**Point 14:** L 119: Pool samples

**Response 14:** Revised as requested.

**Point 15:** L 131: at which stage?

**Response 15:** Sorry for the misinterpretation. By saying “at this stage”, I meant that an infection rate composed 95% among infected individuals (S. sitiens).

**Point 16:** Decrease of titer is in contrast to Rochon, Baker, who, due to the reference cited, examined Porcine Reproductive and Respiratory Syndrome Virus. This should be clarified in the text.

**Response 16:** Clarified as requested.

**Point 17:** Use of “in this study” is misleading when describing the present study

**Response 17:** Revised as requested.

**Point 18:** Reference for impact and description of the mentioned impact of the alimentary tract on LSDV is missing

**Response 18:** Revised as requested.

**Point 19:** Again: Schurrer, Dee also examined porcine reproductive and respiratory syndrome virus, this should be mentioned to be clear

**Response 19:** Revised as requested.

Response to Reviewer 3 Comments

**Point 1:** ) Although LSDV was isolated from all three stomoxys species, peak virus titer was found at 0h and quickly decreased within 24h, indicating the LSDV failed to replicate in stomoxys species. The author previous published paper demonstrated the transmission of LSDV by three stomoxys species under laboratory condition. These species may not transmit LSDV on natural condition.

**Response 1:** It is a well-known fact that transmission of LSDV from diseased to a healthy animal does not occur in their close contact in the insect secure facilities. Moreover, viable LSDV was isolated from these species’ proboscises. Under field conditions, a large number of flies that feed on the erupted lesions must carry or at least become contaminated with pathogen during the feeding process. Given the fact that cattle can be easily infected with LSDV using an intradermal method of inoculation leads to the suggestion of insect vector involvement since insects introduce virus into their hosts in a similar manner.

**Point 2:** In addition to virus retention, the virus transmission by these stomyxys species, which is more interesting aspect should be included in this manuscript. Unfortunately, the virus transmission was published by author.

**Response 2:** I am truly sorry for not being able to address this point as transmission study has already been published. To make a manuscript more saturated, we added statistical analysis using SAS software.
